# Bloodstream Infection in the Intensive Care Unit: Evolving Epidemiology and Microbiology

**DOI:** 10.3390/antibiotics13020123

**Published:** 2024-01-26

**Authors:** Carly Munro, Marya D. Zilberberg, Andrew F. Shorr

**Affiliations:** 1 Medstar Washington Hospital Center, Washington, DC 20010, USA; carly.munro@medstar.net; 2 Evimed Research Group, Goshen, MA 01032, USA; evimedgroup@gmail.com

**Keywords:** antibiotic, bacteremia, bloodborne infection, intensive care unit, outcomes, resistance

## Abstract

Bloodstream infections (BSIs) arising in the intensive care unit (ICUs) present a significant challenge and we completed a narrative review of the emerging literature on this issue. Multiple reports document that these infections are associated with substantial morbidity and mortality. Also, they can be caused by a variety of pathogens. Generally classified as either community or hospital in onset, or as either primary or secondary in origin, the microbiology of ICU BSIs varies across the globe. Gram-positive pathogens predominate in certain regions such as the United States while Gram-negative organisms occur more frequently in Europe, Asia, and Latin America. The incidence of ICU BSIs climbed during the recent pandemic. BSIs complicating the care of persons suffering from Severe Acute Respiratory Syndrome Coronavirus-2 (SARS-CoV-2) infection significantly heighten the risk for death compared to patients who develop ICU BSIs but who are not infected with SARS-CoV-2. Furthermore, rates of antimicrobial resistance are generally increasing in ICU BSIs. This fact complicates attempts to ensure that the patient receives initially appropriate antimicrobial therapy and is of particular concern in Methicillin-resistant *Staphylococcus aureus*, Carbapenem-resistant Enterobacterales, and *Acinetobacter baumannii*. Fortunately, with respect to clinical application, preventive measures exist, and recent analyses suggest that increased collaboration between infectious disease specialists and intensivists can improve patient outcomes.

## 1. Introduction

Bloodstream infection (BSI) presents a major challenge in the critically ill. Formally, a BSI represents the bacterial invasion of the normally sterile bloodstream. Clinically, a combination of blood culture positivity and appropriate clinical signs and symptoms of infection define a BSI. BSIs diagnosed in the intensive care unit (ICU) remain associated with substantial morbidity and mortality. For example, multiple analyses indicate that BSIs in the ICU add substantially to both length of stay (LOS) and cost [1,2]. For example, Taddei et al. recently noted that crude mortality rates for ICU BSIs exceed 40% [3]. Moreover, Allel and colleagues estimated that added costs related to an ICU BSI exceed USD 30,000/case [4]. Conversely, many individuals suffering from BSIs are severely ill. Investigators estimate that between 30 and 40% of BSIs result in either sepsis or septic shock [1,2]. The increasing prevalence of antimicrobial resistance (AMR) encountered both in the hospital, generally, and in the ICU, specifically, further complicates the care of patients with BSIs. As the prescription of timely and in vitro active antibiotic therapy is a central determinant of outcomes in BSIs, growing rates of AMR make it difficult for clinicians to ensure that those with BSIs receive initially appropriate antimicrobial therapy. For BSIs arising as a complication of infections elsewhere, urgent source control represents an additional key objective and cannot be delayed.

Patients in the ICU face a particularly heightened risk for BSI. Their general severity of illness, for instance, can result in a relative degree of immunosuppression and make them prone to multiple types of infection, including bacteremia [1,2,3,4,5]. Likewise, many ICU interventions increase the potential for a BSI. Examples of such processes and procedures range from surgery and mechanical ventilation to the utilization of continuous renal replacement therapy and extracorporeal membrane oxygenation. The need for intravascular catheters (CVCs), though, represents the most significant variable that increases the chance for a BSI in the ICU [4,5].

Though several recent reviews suggest key principles for both the diagnosis and treatment of BSIs, none have reviewed recent reports discussing both the evolving epidemiology and microbiology of these important infections [1,2,3]. Newer analyses also have explored the impact of the Severe Acute Respiratory Coronavirus-2 (COVID-19) pandemic both on the timing of onset of BSI in the ICU and the most frequent culprit pathogens isolated. These reports indicate that the pandemic has had a distinct impact on the microbiology of BSIs in the ICU. Other more contemporary studies have also focused on BSIs caused by select bacteria associated with high rates of AMR and mortality. Specifically, important pathogens that require discussion range from carbapenem-resistant Enterobacterales (CRE) and *Acinetobacter baumannii* (AB) to methicillin-resistant *Staphylococcus aureus* (MRSA). Therefore, we sought to review these topics so as to provide clinicians with a comprehensive perspective on ICU BSIs.

In short, we sought specifically to conduct a narrative review based on select key studies on the general epidemiology and microbiology of ICU BSI published since 2019. We also aimed to examine, summarize, and comment on larger analyses relating to COVID-19 and BSI risk while also reviewing several recent reports (mainly since 2019) addressing specific difficult to treat (DTR) pathogens in ICU BSI.

## 2. Classification Schemes

BSIs can be classified in different ways [1,2,5]. Epidemiologically, researchers often categorize BSIs as community onset (CO) or as hospital-acquired (HA). Alternatively, BSIs are frequently described as either primary or secondary. In secondary infections, the BSI arises as a complication of infection elsewhere (i.e., pneumonia, cellulitis, etc.). In primary BSIs, physicians cannot locate an alternative site of infection. The specific microbiology of such BSIs can differ based on these factors. Though Enterobacterales predominate in most situations because of their general prevalence, the frequency of important pathogens such as *Pseudomonas aeruginosa* (PA) and *Staphylococcus aureus* (SA) will vary based on the primary infection site and patient location of onset (e.g., CO vs. HA).

Central-line-associated BSIs (CLABSIs) are a unique type of BSI. Bacteremias associated with CVCs have a distinct evolution and reflect a direct complication of the use of invasive, indwelling devices [5,6]. In this vein, and in light of the multiple approaches available for preventing this potentially severe event, CLABSI rates serve as a key metric of quality and safety [6,7,8]. Arterial lines may also result in a BSI, although this occurs far less frequently than with traditional CVCs. Finally, BSIs can be described as either complicated or uncomplicated. In complicated BSIs, often there is a non-removable source of infection or, alternatively, a distal metastatic complication. Metastatic complications most often occur in the setting of SA bacteremia but may be seen with other organisms.

## 3. Epidemiology and Microbiology

Understanding the evolving epidemiology and microbiology of ICU BSIs is crucial both for the prevention and treatment of these infections. A thorough appreciation of these issues can also help physicians design antibiotic protocols that better ensure that patients receive initially appropriate antibiotic regimens. It is crucial to acknowledge that the epidemiology and microbiology of BSIs vary across the globe. Both variability in the incidence of important endemic pathogens and the heterogeneity in healthcare organization and delivery across different health systems affect the types of BSIs seen in different ICUs. Thus, one cannot presume that observations from a specific nation apply to other countries. In turn, it is necessary to explore, in detail, more regional data based on analyses not only from the United States but also from Europe and from other parts of the globe. Table 1 summarizes key aspects of the key recent studies.

From a US vantage point, in a retrospective cohort study, Gouel-Cheron and co-workers, as noted in Table 1, explored BSI in over 150,000 patients in the ICU across 85 hospitals [9]. Among their cohort, 6906 patients were diagnosed with a BSI while in the ICU and most (approximately 80%) were CO. The remainder (n = 1306) arose while the patient was hospitalized in the ICU [9]. Although the median time to HA ICU BSI equaled 6 days, some developed as early as four days after ICU admission. Consistent with earlier reports, these researchers found that more than 70% of BSIs represented secondary infections. Although patients with a BSI while in the ICU were critically ill, disease severity as measured with the Sequential Organ Failure Assessment (SOFA) score did not differ between subjects with CO and HA BSIs. Microbiologically, Gram-positive organisms predominated with SA accounting for 20% of all ICU BSIs (Table 1). Amongst Gram-negative pathogens, *Escherichia coli* was most frequent and seen in approximately 15% of blood cultures. PA and AB were isolated in fewer than approximately 3% and 1% of subjects, respectively. The specific distribution of bacteria differed though based on the location of infection onset. Gouel-Cheron et al. confirmed that Gram-negative organisms occur more often in HA as opposed to CO BSIs [9]. Specifically, Gram-positive organisms accounted for only a third of HA BSIs. Additionally, PA was seen twice as often in HA infection (i.e., 5% of HA BSIs vs. 2.5% of CO BSIs), and although AB was noted rarely in both CO and HA BSIs, it was isolated very rarely in CO processes. Similarly, AMR was more of an issue in ICU-onset BSIs than in CO infections. Nearly a third of Gram-negative HO BSIs demonstrated AMR compared to less than 10% in CO BSIs. Not surprisingly, both MDR Gram-positive and Gram-negative pathogens arose more often in ICU-acquired BSIs as well.

In an analysis exploring variables associated with ICU BSI, these authors concluded that demographic, institutional, and treatment variables increase the risk for an ICU BSI. Many of the factors identified with ICU-onset BSI have been identified previously such as the ICU type, severity of illness, prior exposure to antimicrobials, use of CVCs, and duration of mechanical ventilation. While the report by Gouel-Cheron et al. provides important information on ICU BSIs and is particularly important given its sample size, their study does have substantial limitations [9]. The utilization of large administrative datasets, like the one these investigators relied upon, is prone to coding bias (in which patient syndromes are misclassified) and lacks important patient level information—such as the results of routine laboratory testing and certain processes of care factors. They also give little insight as to which BSIs are primary vs. secondary in origin. Irrespectively, this report sheds light on the current state of ICU BSIs from a US perspective [9].

Moving to a European perspective, Tabah and colleagues analyzed the microbiology of ICU BSIs across 52 nations participating in a prospective observational study under the auspices of both the European Society of Intensive Care Medicine and the European Society for Clinical Microbiology and Infectious Diseases (EUROBACT-2) [10]. The prospective nature of this project (Table 1) and its focus on HA BSI differentiate it from the report of Gouel-Cheron et al. [9,10]. The time to onset of these HA BSIs in this European cohort was longer than that reported in the US (e.g., 13 days vs. 6). Conversely, a primary BSI accounted for nearly one in seven infections—which is similar to what is described in the US. The general distribution of pathogens was also similar. Among these European patients who were critically ill with HA BSI, Gram-negative organisms predominated [10]. Reflecting important differences in the prevalence of select endemic MDR organisms on different sides of the Atlantic, more than 20% of patients examined by Tabah and co-workers were infected with an *Acinetobacter* spp. (Table 1). In the US, these bacteria, as noted above, are rarely isolated. On the other hand, MRSA was a relatively uncommon bacteria in BSIs in European ICUs. The findings of Tabah et al. confirm the substantial mortality associated with ICU BSIs: nearly a third of subjects died while in the ICU [10].

Tabah et al. extended the significance of their work by conducting a careful analysis of predictors of mortality in ICU BSI [10]. Severity of illness and age, not unexpectedly, independently correlated with mortality. The presence of a difficult-to-treat Gram-negative bacteria was also linked with death. One modifiable process of a care variable stood out in relation to mortality: infrequent consultation with a clinical pharmacist independently increased the risk for death by approximately 30%. The major impact of failing to engage with experts in antimicrobial dosing and monitoring identifies (1) the value these individuals provide and (2) identifies an important pathway forward for improving patient outcomes. This previously unexplored relation between consulting a clinical pharmacist and mortality in the setting of HA BSI in the ICU reinforces the value of a prospective analytic paradigm like that utilized in EUROBACT- 2. This type of crucial process of a care factor cannot be studied in large administrative databases like the one relied upon by Gouel-Cheron et al. [9].

In another multicenter, prospective report from Europe, Perez-Crespo and colleagues confirmed the insights gleaned from EUROBACT-2 [11]. Specifically, the PRO-BAC study described the epidemiology of healthcare-associated BSIs across Spain. Although narrower in geographic scope, this analysis was conducted over 6 months in 2016–2017 and enrolled over 6000 subjects with BSIs across 26 hospitals (Table 1). Approximately one-third represented HA processes [11]. A quarter of HA BSIs were seen in the ICU and were associated with sepsis and septic shock. In contrast to the findings of others, more than 30% of all ICU BSIs were classified as CLABSIs. Urinary tract infections and intra-abdominal infections followed as likely secondary sources while nosocomial pneumonia (NP) was an infrequent cause of a BSI. This is a contrast to larger and more geographically broad case series in which NP represents the most common cause of secondary BSIs—again indicating the importance of understanding local epidemiologic patterns [1,2,6]. Additionally, in contrast to the findings of Gouel-Cheron et al., the breakdown in pathogens in ICU-onset BSIs was more evenly divided between Gram-positive and Gram-negative bacteria (Table 1)—as was seen also in EUROBACT-2 [9,10]. Gram-positive organisms accounted for 41% of BSIs while more than half represented Gram-negative organisms. At the same time, analogous to the two larger studies described above, Perez-Crespo et al. encountered AMR pathogens more often in HA ICU BSIs [11]. For example, extended-beta lactamase production was observed in 17.5% of isolates arising in the ICU vs. only 7% of CO BSIs. Carbapenem resistance among Enterobacterales occurred rarely (4%) but was still more frequent with HA infections than with CO infections.

Beyond Europe and the US, little systematic data exist describing ICU BSI epidemiology. One recent survey, however, exploring 24 countries presented information gleaned from not only several high income (HI) European nations but also countries classified as upper middle income (UMI) and lower middle income (LMI) [12]. As such, more than half of the participating nations in this analysis (n = 16) are located in the Middle East, South Asia, and Latin America. Only approximately 20% of the 771 cases of BSI evaluated came from HI countries. In HI and UMI sites, HA BSIs predominated. In LMI ICUs, CO infections occurred as frequently as HA BSIs. Although this pattern varies from that seen in most of Europe, it appears akin to what Gouel-Cheron and co-workers reported for the US [9,11]. This difference may reflect methodological nuances in data collection. However, it more likely shows the importance of local and organizational factors in driving the epidemiology of ICU BSI. Readers therefore should interpret the results of studies on this topic with caution as their findings may or may not be generalizable to their particular institution. Despite this caution, El-Sokkary et al. noted that MDR occurred significantly more often in HA than CO ICU BSIs [12]. This theme has been seen consistently across the multiple analyses described thus far.

In short, contemporary descriptions of the epidemiology and microbiology of ICU BSIs reveal that these infections remain associated with substantial morbidity and mortality. More importantly, the distribution of specific pathogens and antibiotic resistance patterns varies across the globe. No one organism systematically predominates. Nonetheless, MDR is seen more often in HA than in CO BSIs, irrespective of geography.

## 4. COVID-19 and ICU BSI

The ongoing pandemic has had a profound impact on ICU care and outcomes. During various phases of the pandemic, up to 20% of patients with an acute COVID-19 infection required ICU admission and mechanical ventilation. These factors placed many ICUs and the staff who supported them under dire strain. Consequently, infection control measures suffered and nosocomial infections became a major challenge. In fact, one report suggests that nearly half of all deaths in the ICU from COVID-19 were caused by NP [13]. As a consequence of these pressures, many surveillance studies demonstrate an alarming increase in the rates of AMR across the globe. The impact of the pandemic on ICU BSIs, though, is less clear. Three well-done analyses (see Table 2) from Europe have specifically investigated this question.

First, Buetti and colleagues conducted a case–control study on previously prospectively collected data across multiple French ICUs [14]. They specifically hypothesized that COVID-19 infection would increase the risk for an ICU BSI. Drawing on a cohort of over 1800 patients cared for in six ICUs, they subsequently matched 235 patients without COVID-19 to a similar number of subjects with COVID-19. Specifically, the investigators matched for ICU, admission type, age, and severity of illness. Fewer than 5% of individuals without COVID-19 developed an ICU BSI compared to 15% of those with COVID-19 (Table 2, *p* < 0001). Median time to BSI onset in the COVID-19 cohort equaled 12 days, a notably late onset relative to the ICU BSIs detailed in some of the broader database studies discussed earlier [9,10,11]. In fact, these authors observed that the risk for ICU BSI in COVID-19 cases increased substantially after day 7. Reflecting that more than 20% of the BSIs associated with COVID-19 were deemed to be CLABSIs, the most common organisms in this population were coagulase-negative Staphylococci and Enterococci [14]. The nexus between time to onset and infection type suggests that physicians must remain vigilant in central line care for those with respiratory failure from COVID-19. Though CLABSIs are generally infrequent phenomena, these data show that in COVID-19, if a new fever develops, accompanied by clinical deterioration, physicians should contemplate presumptive CVC removal and empiric therapy to cover pathogens associated with CLABSIs.

Second, Massart et al. explored the microbiologic and prognostic aspects of ICU BSIs in COVID-19 [15]. Unlike the report by Buetti et al., as noted in Table 2, these researchers completed a secondary analysis of the prospective European “COVID-ICU” study [11,12]. Because the data were collected specifically to understand COVID-19 in the ICU, they provide a more precise estimate of the true risk of ICU BSI in COVID-19. Thus, among 4010 patients, 19.5% had positive blood cultures during their ICU stays (Table 2). These investigators calculated that 10.3 BSIs occurred per 1000 patient days [15]. To place this number in perspective, in many reports, a CLABSI rate of 2 per 1000 line days is considered excessive [1,2]. Corroborating the results of Buetti et al., the median time to onset for ICU BSI was 9 days [15]. Importantly, these authors did not notice an increased risk for BSI with use of either anti-inflammatory medications or corticosteroids (Table 2). Patients diagnosed with an ICU BSI were not only more likely to die but also had longer ICU LOSs and had fewer ventilator-free days.

Third, relying on the EUROBACT-2 data described earlier, a different group of researchers aimed to assess the epidemiology and outcomes for ICU BSIs associated with COVID-19 [16]. For this analysis, 53 centers contributed a median of 10 patients each. In the end, the study included 829 patients with ICU BSIs of whom 30.4% had a severe COVID-19 infection (Table 2). Researchers classified roughly 80% of BSIs as secondary to infection elsewhere. Although CLABSIs occurred frequently in patients with COVID-19, there was no difference in the prevalence of CLABSI between subjects with and without COVID-19 [16]. This finding (see Table 2) differs from the results of Buetti et al. noted earlier [14]. Also distinct from the conclusions of Buetti et al. and of Massart et al., the time to BSI onset was similar between those with and without COVID-19 infection. Specifically, in both populations, the median interval from admission to the BSI diagnosis was approximately 14 days [16]. Furthermore, Gram-positive bacteria caused relatively more BSIs and MDR Gram-negative ones caused relatively fewer BSIs in those suffering from COVID-19. This could reflect differences in the rate of antibiotic exposure between the two arms—as those without COVID-19 were less frequently exposed to antimicrobials. In other words, the higher BSI frequency due to Gram-positive pathogens may have arisen as a consequence of selection pressure. The potential for selection pressure to drive this observation is also consistent with the fact that MDR Gram-negative bacteria arose more often in subjects in the COVID-19 arm.

Irrespective of differing observations regarding select aspects of COVID-19 ICU BSI epidemiology and microbiology, these authors documented that BSIs in the setting of COVID-19 result in poor outcomes (Table 2). Patients with both COVID-19 and an ICU BSI were nearly twice as likely to die in the short term as opposed to those without COVID-19 but with an ICU BSI. Mortality rates were even higher in persons with MDR Gram-negative BSIs and COVID-19—nearly 85% of these subjects expired by day 28 [16].

What general conclusions can be drawn regarding the impact of the pandemic on ICU BSIs? It appears that those with COVID-19 are more prone to ICU BSIs, even when one corrects for issues related to the extended duration of time those with COVID-19 spend in the ICU. It is unclear whether it is because viral infection facilitates the invasion of bacteria into the bloodstream or because of selection pressure from antibiotic exposure promoting superinfection—or any number of other factors. Untangling the interaction of these factors will prove to be challenging. However, in one sense, the cause of the relationship between COVID-19 and BSI risk is unimportant in so far as, irrespective of the mechanism, multiple studies underscore that clinicians must remain vigilant against the chance for bacteremia in these patients. Likewise, BSI in subjects with COVID-19 results in horrid outcomes. To improve mortality in this scenario, physicians must act aggressively to ensure that the patient receives initially appropriate antibiotic therapy and strive to ensure that source control is achieved.

## 5. Multi-Drug-Resistant Organisms

As noted earlier, the rising incidence of AMR continues to complicate the care of those with ICU BSI. Recent studies focusing on CRE, AB, and MRSA make evident the variability in the epidemiology of these pathogens across the globe—for some nations, these pathogens are of a major concern while in others they have not evolved into commonly encountered bacteria. Nonetheless, one theme is consistent across the various MDR BSI. In many cases, BSIs caused by these MDR pathogens are highly lethal.

### 5.1. Carbapenem-Resistant Enterobacterales

The SENTRY Antimicrobial Surveillance Program collected BSI data from over two hundred medical centers, internationally, between 1997 and 2016. This report was not limited to patients who were in ICUs although many patients required critical care services. These investigators demonstrated that multi-drug resistance among Enterobacterales increased from 6.2% in 1997 to 15.8% by the end of the surveillance period. While the prevalence of CRE varied across the globe, it was highest in Latin America (28.1%) [17].

In a similar study undertaken at 25 large centers in China, the incidence of CRE was approximately 40 cases per 100,000 hospital admissions, while in similar studies in the United States and Europe, the rates of CRE bacteremia were significantly lower at nearly 1.3 per 100,000 and 2.9 per 10,000 hospital admissions, respectively [18,19,20,21,22]. Again, these findings illustrate that “geography matters”. The CRE phenotype was largely seen in *Klebsiella pneumoniae* (73.9%), followed by *Escherichia coli* (16.6%). Notably, the mortality rate for patients with a CRE BSI was a shocking 43.1% [19].

Despite the variability in the epidemiology amongst CRE BSIs in the ICU, the mortality rate related to this infection, as alluded to above, remains substantial. Confirming this, a recent observational study in the US demonstrated that 38% of patients with CRE bacteremia developed septic shock. This study noted a 30-day mortality rate of 49%, similar to what has been noted in other sites outside the US [23]. Contributing to the high mortality rate, these researchers noted that the median time from onset of bacteremia to appropriate antimicrobial coverage was 47 h. This is consistent with the observation that AMR BSIs are more likely to result in initially inappropriate therapy and, consequently, lead to a day of effective treatment [1,2].

In summary, the incidence of CRE BSI is increasing at a significant pace and is associated with high mortality rates. There remains dramatic geographic heterogeneity in the incidences of CRE. Given the potential for significant delays in the initiation of adequate antimicrobial coverage, clinicians must remain vigilant.

### 5.2. Acinetobacter

The incidence of AB also varies across geographic regions, much like the case of CRE. As previously mentioned, AB was rarely isolated in the studies performed in ICUs in the United States but was seen commonly (20.3%) in EUROBACT-2 sites [9,24]. In an international study, AB was found to have a worldwide incidence of 2%, with higher incidences noted in Latin America and Asia (4.2 and 3.2%, respectively) [17]. Additionally, MDR rates were the highest in AB of any pathogen studied, demonstrating an MDR rate of 70.6% and a pan-resistance rate of 0.9% [17,24].

Furthermore, in a recent retrospective study of AB BSI, Alenzai et al. reviewed their experience with this pathogen during both the pre-COVID-19 and pandemic eras [25]. The majority of cases occurred in patients who were critically ill. Irrespective of whether associated with COVID-19 infection or not, survival rates were abysmal. Fewer than 30% of subjects lived to hospital discharge. The average length of stay in both groups exceeded 3 weeks. These poor outcomes likely reflect that all AB isolated were MDR.

Outcomes for patients are even worse, not surprisingly, if the AB is resistant to colistin. For example, in a review of 13 patients with colistin-resistant AB, two-thirds presented septic shock and all died [26].

In order to gain insight into risk factors for an ICU BSI with AB, a recent multicenter case–control study compared patients with AB BSIs to matched patients with AB infections not complicated by a BSI. Significant risk factors for BSI included being immunocompromised, admission to the ICU prior to a positive blood culture, and presence of a central nervous system infection [27]. Additionally, the 30-day mortality was higher in patients with a BSI than those with infections present at other sites (34% vs. 21%) [27]. Just as in other studies, AB demonstrated the highest incidence of resistance to existing antimicrobial therapies, with concerning trends indicating increasing rates of pan-resistant AB.

### 5.3. Methicillin-Resistant Staphylococcus aureus

MDR is not only a concern for Gram-negative organisms but is also a worry in select Gram-positive bacteria, namely SA. Diekema and colleagues focusing on the US demonstrated an alarming increase in the incidence of MRSA BSIs in the initial years of their surveillance, pealing in the period from 2005 to 2008 (from 33.1% to 44.2%) [28]. However, in the later portion of their surveillance period, the incidence of MRSA began to decrease. This trend is consistent with other recent studies and correlated temporally with increased emphasis on widespread infection prevention efforts. Unfortunately, the enrollment in this analysis was not limited to patients who were critically ill, and therefore the generalizability of their findings to the ICU is uncertain.

From a US perspective, Ham and co-workers investigated recent risk factors for an MRSA BSI [29]. Approximately half of their cohort developed the index bacteremia while in the ICU. Not surprisingly, most patients with ICU MRSA BSI had either undergone surgical procedures or had indwelling devices. Consistent with results noted earlier, most MRSA BSIs in the ICU reflected a complication of central venous catheterization while the remainder arose in the setting of a primary infection elsewhere (e.g., pneumonia).

A national observational study undertaken in 139 ICUs in the United Kingdom, interestingly, monitored the incidence of MRSA bacteremia for a ten-year period (2007–2017) after the implementation of infection control measures such as mandatory reporting of infections with specific organisms and a national education campaign, along with the dissemination of evidence-based guidelines for prevention. At the initiation of this study, MRSA accounted for 12% of all BSIs, including those diagnosed in the ICU. However, at the end of the monitored time period, MRSA accounted for fewer than 2% of BSIs [30]. These results reveal that preventive measures can reduce the incidence of select MDR ICU BSIs. Why such strategies have proven ineffective at addressing MDR Gram-negative BSIs remains uncertain.

## 6. Conclusions

Multiple studies document the growing prevalence of ICU BSIs and their variable microbiology from country to country. Certain MDR pathogens, such as MRSA, remain more prevalent in the US while MDR Gram-negative bacteria such as AB and CRE are of greater concern in Asia and parts of Europe and Latin America. Irrespectively, ICU BSIs can be encountered either as a community-onset infection or as a nosocomial complication. Likewise, these infections often are found secondary to infectious processes beginning elsewhere. Despite this heterogeneity in the epidemiology and microbiology of ICU BSIs, they remain associated with substantial morbidity and mortality.

What can clinicians do to improve outcomes for patients who are critically ill with a BSI? First, they must emphasize prevention. Clearly, as described above, preventive protocols have proven effective, especially for MRSA. Second, adopting antimicrobial stewardship principles that limit the exposure of patients to unneeded antibiotics can help to reduce the selection pressure that drives AMR. Rapid diagnostics can also aid in this sense by driving clinicians to narrow antibiotics when they are not needed. Additionally, treatment protocols that rely on the raft of evidence that documents that shorter courses of antibiotics are as effective as longer durations must be implemented as part of comprehensive initiatives to address AMR in ICU BSIs.

Finally, emerging evidence shows that enhanced collaboration between physicians primarily responsible for patients with ICU BSIs and experts in infectious diseases can reduce mortality. For example, Shulder and colleagues in an analysis of nearly 5000 patients with a Gram-negative BSI documented that patients who were seen by infectious disease specialists were 40% more likely to survive, after adjusting for some select confounders [31]. A similar analysis by Ramanathan et al. addressing only *Pseudomonas aeruginosa* confirmed a similar impact of including infectious disease experts in the care of those with BSIs [31]. Readers should note that the majority of subjects in these reports were never cared for in an ICU. Additionally, the retrospective nature of these analyses limits their conclusions. Nonetheless, there is every reason to foster collaboration as the care for patients with ICU BSIs becomes more and more complex. In the end, it will take a multifaceted approach based on validated interventions to improve outcomes for those with ICU BSIs.

## Figures and Tables

**Table 1:** Recent General Studies of Bloodstream Epidemiology.

Authors	Reference	Study Name	Years Studied	Methodology	Geography	Subjects	Most Common Pathogens in ICU BSI	Findings of Note
Gouel-Cheron A., et al.	[9]	NR	2009–2015	Observational, retrospective, administrative database	US	A total of 150,948 patients in the ICU	*S. aureus*, *S. pneumoniae*, *E. coli*	In total, 4.2% of patients in the ICU suffered a BSI, 12% arose in the ICU
Tabah A., et al.	[10]	EUROBACT-2	2019–2021	Observational, prospective, cohort	Global except for US	A total of 2600 patients in the ICU with hospital-acquired BSI treated in the ICU	*Klebsiella* spp., *Enterococcus* spp., *S. aureus*, *P. aeruginosa*	The median time to appropriate antibiotic therapy was 1 day in BSIs due to generally susceptible pathogens vs. 4 days in cases of DTR infections
Perez-Crespo P.M.M., et al.	[11]	PROBAC	2016–2017	Observational, prospective, cohort	Spain	A total of 6345 hospitalized patients with BSI (1708 subjects with severe sepsis or septic shock)	*E. coli*, *S. aureus*, *Klebsiella* spp.	Most common secondary sites for ICU BSIs were CVCs and the urinary tract

Abbreviations: BSI—bloodstream infection, CVC—central venous catheter, DTR—difficult to treat, ICU—intensive care unit, NR—not reported.

**Table 2:** Summary of Key Studies Investigating COVID-19 and Bloodstream Infections in the Intensive Care Unit.

Authors	Reference	Years	Study Design	Geography	Subjects	Prevalence of BSI in COVID-19	Notable Observations
Buetti N., et al.	[14]	2020	Retrospective case control	France	A total of 321 subjects with COVID-19, 1029 controls without COVID-19	- 14.9% vs. 3.4% (control)	- BSI risk in COVID-19 increases substantially after day 7 - 30-day survival worse in COVID-19 with ICU BSI
Massart N., et al.	[15]	2020	Secondary analysis of prospective observational study	International	A total of 4010 patients with COVID-19 cared for in the ICU	- 19.5% of patients with COVID-19 - 10.3 BSIs per 1000 patient days	- Median time to BSI: 9 days - BSI increases risk for death - Tociluzimab did not increase risk for BSI
Buetti N., et al.	[16]	2019–2021	Secondary analysis of prospective observational study	International	A total of 829 patients with BSI (30.4% with COVID-19)	- NR	- No difference in time to BSI between patients with and without COVID-19 - DTR pathogens occurred more often in COVID-19 BSI

Abbreviations: BSI—bloodstream infection, DTR—difficult to treat, ICU—intensive care unit, NR—not reported.

## Data Availability

Not applicable.
